# Neck-Specific Exercises With Internet Support Versus Standard Neck-Specific Exercise for the Treatment of Chronic Whiplash-Associated Disorders: Cost-Effectiveness Analysis

**DOI:** 10.2196/88832

**Published:** 2026-10-28

**Authors:** Swarupa N Kshirsagar, Anneli Peolsson, Gunnel Peterson, Koustuv Dalal

**Affiliations:** 1 Department of Public Health Science School of Health Sciences Mid Sweden University Sundsvall, Västernorrland Sweden; 2 Unit of Physiotherapy Department of Health Medicine and Caring Sciences Linköping University Linköping, Östergötland Sweden; 3 Unit of Clinical Medicine Occupational and Environmental Medicine Centre, Department of Health Medicine and Caring Sciences Linköping University Linköping, Östergötland Sweden; 4 Centre for Clinical Research Sörmland Uppsala University Eskilstuna, Uppsala Sweden; 5 University of Johannesburg Johannesburg, Gauteng South Africa

**Keywords:** whiplash injuries, exercise therapy, internet-based intervention, spine, cost-effectiveness

## Abstract

**Background:**

There is currently scarce information about the cost-effectiveness of interventions for individuals with chronic whiplash-associated disorders (WAD) or of internet-based exercise programs for neck pain, including WAD.

**Objective:**

This study aimed to investigate the cost-effectiveness of a 3-month neck-specific exercise program with internet support in combination with 4 physiotherapy visits (neck-specific exercise with internet support [NSEIT]) compared with the same exercises performed twice a week (24 times) at a physiotherapy clinic (neck-specific exercise [NSE]) for individuals with chronic WAD.

**Methods:**

This was a prospective, multicenter, randomized controlled trial of the cost-effectiveness of the interventions among 140 individuals with chronic moderate to severe WAD with 3- and 15-month follow-up. The main outcome was the incremental cost-effectiveness ratio (ICER).

**Results:**

At the 15-month follow-up, the total costs calculated for NSEIT and NSE were SEK 12,882 (95% CI 17,406.30-8358.42) and SEK 40,087 (95% CI 51,764.32-28,410.96), respectively (SEK 1=US $0.10 as of October 6, 2026). The health-related quality of life (HRQoL) score at 15 months was 0.662 (95% CI 0.73-0.59) and 0.711 (95% CI 0.77-0.65) for NSEIT and NSE, respectively. The ICER for HRQoL was SEK 555,209.80 per point when the NSEIT group was compared with NSE. The internet-based intervention was less costly and less effective than NSE at the 15-month follow-up. The cost-effectiveness acceptability curve showed that at a willingness to pay threshold of SEK 500,000 per point, there was a moderate probability (0.57) of the intervention being cost-effective with a loss of HRQoL. One-way sensitivity analysis showed that the health care costs of individuals receiving NSE and NSEIT affected the ICER.

**Conclusions:**

NSEIT intervention—an eHealth intervention for chronic WAD—was less costly and less effective compared with NSE. Health care costs associated with both interventions affected the ICER; therefore, patients’ overall well-being should also be considered when treating chronic WAD. Even though the cost effectiveness acceptability curve showed that the cost savings from NSEIT outweighed the lost health outcomes, the reliability of the findings was limited by the exclusion of presenteeism costs, patients’ self-reported health care use, and the small difference in health outcomes.

**Trial Registration:**

ClinicalTrials.gov NCT03022812; https://clinicaltrials.gov/study/NCT03022812

## Introduction

Whiplash-associated disorders (WAD) are often the result of rear-end motor vehicle crashes, where the individual experiences acceleration and deceleration leading to indirect injury to the neck [1,2]. Whiplash injuries represent the most prevalent form of trauma, affecting approximately 43.8% of individuals involved in motor vehicle collisions [3]. Such injuries tend to be chronically debilitating and expensive. Approximately 50% of individuals with WAD might continue to endure neck pain and disability [3-5]. Additional symptoms include blurred vision, fatigue, unsteadiness, and dizziness, which significantly impact quality of life [1,6,7]. There are significant health care costs associated with chronic symptoms for more than 6 months and subsequent productivity loss for individuals with WAD [8,9].

Rehabilitation through supervised neck-specific exercises (NSE) is an integral part of the treatment of WAD that helps with pain and functionality [10]. The literature indicates that internet-based interventions have efficacy comparable to or better than that of traditional exercise-based treatments for various musculoskeletal disorders [11-14]. While the literature on internet-based interventions for WAD is limited, studies and reviews have assessed the effectiveness of internet-based physiotherapeutic interventions for patients with musculoskeletal disorders.

Internet-based interventions are referred to as eHealth, telehealth, digital health, and mobile health interventions. Internet-based physiotherapeutic interventions typically combine exercise programs with educational content delivered through digital platforms, including exercise videos, images, written instructions, and information [15,16]. Internet-based treatments have been reported to reduce costs, facilitate patient education, and support evidence-based primary care [17,18].

Internet-based interventions targeted at musculoskeletal disorders are often developed to improve mobility, strengthen patient autonomy, and enhance the ability to maintain long-term self-care habits [19]. These interventions may include remote physiotherapy, online or telephonic consultations with rehabilitation professionals [20,21], and web-based support for self-management exercises [22]. The effects of internet-based interventions aiming at rehabilitation have been documented for joint replacement, osteoarthritis, and back pain; however, the outcomes were statistically insignificant when compared with in-person rehabilitation methods [23]. Earlier studies comparing e-exercise and standard physiotherapy for osteoarthritis [24] and chronic lower back pain [25] found that the eHealth interventions were neither effective nor cost-effective from societal or health care perspectives, and total societal and health care costs did not differ significantly between groups. A study that adopted a more comprehensive intervention, focusing on patient information about self-care and exercises, was less costly but did not yield significant cost savings [26]. Earlier literature has repeatedly noted that internet-based interventions are severely understudied, particularly with regard to their long-term effects, mechanisms of change, real-world benefits [27], and cost-effectiveness [28]. Another review assessing telehealth rehabilitative programs found that remote exercise programs and virtual consultations contributed significantly to positive patient outcomes for chronic neck pain [20], but it did not review any cost-effectiveness studies of such interventions. One study that included eHealth interventions for neck-related issues, including acute WAD [29], reported that both face-to-face and eHealth interventions resulted reduced pain-related dysfunction. Both interventions were found to be better than standard treatment for providing the individual with educational information (through a pamphlet). Individually tailored physiotherapy treatment for acute WAD was not cost-effective compared with usual care from a health care perspective [9].

eHealth interventions also imply insufficient support, information, and understanding for patients [30] when unsupported by health care providers. NSE with 2 visits per week for 12 weeks for chronic WAD grade II (ie, neck pain and clinical musculoskeletal signs) and grade III (ie, grade II plus neurological signs) was found to be cost-effective from a societal perspective in the treatment of chronic WAD when compared with a behavioral approach or only prescribed physical activity [31]. The combined intervention of internet-based support, along with some visits to health care providers, is preferable for counteracting insufficient support, information, and patients’ understanding [32], especially for chronic WAD.

The current study was based on an internet-based intervention (neck-specific exercise with internet support [NSEIT]) that provided NSE delivered via internet support—offering e-resources for neck exercises for chronic WAD. In an earlier comparative study about the same intervention, neck-specific exercises (NSEIT) were noninferior to the same NSE supervised by a physiotherapist twice a week for 12 weeks [33]. Both groups had a significant reduction in disability and an improvement in health-related quality of life (HRQoL; EQ-VAS) at 15 months of follow-up. EQ-5D-3L improved only in the NSEIT group [33].

Internet-based rehabilitation may reduce treatment costs and physiotherapist time, but economic evaluations are needed to determine whether the balance between costs and effectiveness represents an acceptable trade-off from a health care system perspective. Additionally, limited literature on chronic WAD, its treatment, and the overall dearth of cost-effectiveness studies on internet-based interventions for musculoskeletal disorders further highlight the need for cost-effectiveness assessment while going beyond internet based intervention clinical effectiveness. The current study thus aimed to assess the cost-effectiveness of an internet-based intervention (NSEIT) versus standard NSEs for chronic WAD.

## Methods

### Study Design

This prospective, multicenter, randomized controlled trial compared 2 groups: NSEIT versus supervised NSE with 3- and 15-month follow-ups. The study adhered to the principles outlined in the Declaration of Helsinki. Details of the study protocol and intervention were published elsewhere [30,33,34]. Data were collected from April 6, 2017, to September 15, 2020, and the primary outcome data were published [33]. Written informed consent was obtained from all participants before allocation. The present study was a planned analysis of cost-effectiveness.

### Participants

A total of 140 participants, 70 in the NSEIT group and 70 in the NSE group, with a mean WAD duration of 26 (SD 16.8) months, were included. After advertisements were placed in newspapers, on social media, and on websites, potential participants expressed their interest in participating in the study. They completed a short survey on the study’s website. An experienced physiotherapist interviewed the candidates by telephone regarding their medical history. As the final step in the recruitment process, candidates underwent a physical examination of their neck, conducted by a physiotherapist. If the study criteria were met, participants completed a baseline questionnaire, and an independent test leader conducted physical assessments of the neck.

### Inclusion Criteria

Individuals with a whiplash injury from a traffic accident involving a 4-wheeled motor vehicle at least 6 months ago but less than 5 years ago were included if they had chronic WAD grades II and III [9] verified through clinical examination by the test leader. Additional inclusion criteria were average estimated pain in the last week of at least 20 mm on the visual analog scale [35] neck disability of more than 20% on the Neck Disability Index [36]; working age (18-63 years); daily access to a computer, tablet, or smartphone and the internet; time to follow the treatment program; and neck symptoms within the first week after the injury (ie, neck pain, neck stiffness, or cervical radiculopathy). Answering the baseline questionnaire was a requirement.

### Exclusion Criteria

The exclusion criteria were individuals with signs of head injury at the time of the whiplash injury (loss of consciousness, amnesia before or after the injury, altered mental status such as confusion or disorientation, or focal neurological changes such as changes in smell and taste). An additional exclusion criterion was known or suspected severe physical pathology (including myelopathy, spinal tumors, spinal infection, previous fractures or dislocation of the cervical spine, or ongoing malignancy). Other exclusion criteria were cervical spine surgery, sick leave due to neck pain for more than a month in the year before the current whiplash injury, generalized or more dominant pain elsewhere in the body, other illness or injury that may prevent full participation in the study and/or in which neck exercises are contraindicated, inability to understand and write Swedish, diagnosed severe mental illness (such as psychosis, schizophrenia, or personality disorders), and current alcohol or drug abuse or participation in the earlier NSE study [33].

### Randomization

A computerized block-randomization list, stratified by gender (conducted by a statistician and allocated by a project team member), was used to randomize participants to 2 groups. The randomization was performed by an independent researcher who was not involved in any of the tests or treatments. The researcher sent an opaque envelope containing the patient’s name and contact details, as well as information about the randomization group, to the treating physiotherapist, who invited the patient to a new clinical examination (in accordance with the law) before treatment could start.

Independent physiotherapists provided the treatment in outpatient care. Neither the participants nor the treating therapists could be blinded. The project manager was not involved in the data collection. Participants answered questionnaires at baseline (before randomization), after 3 months (at the end of physiotherapy rehabilitation), and after 15 months (one year after the intervention] [33].

### Intervention

#### NSE

In the NSE group, participants received an explanation and justification for the exercises, including information on the neck’s musculoskeletal anatomy relevant to them. These were to motivate the patients and help make them feel safe and reassured. Elements of a behavioral approach were included, such as education and strategies for dealing with neck pain relapse.

Participants attended physiotherapy clinic sessions twice weekly for 12 weeks to perform prescribed exercises with a physiotherapist (a total of 24 times). They also completed exercises at home, although without access to the internet-based system for training the neck and shoulder muscles. The exercises were individually adjusted according to the individual’s physical conditions and progressively increased in severity and dose. At the end of the treatment period, the participants were encouraged to continue practicing on their own.

#### NSEIT

In the NSEIT group, participants received the same information and exercise modules as the NSE group. This module included only 4 visits to a physiotherapist. The first visit included introducing the exercises, advancing them as needed, and ensuring they were performed correctly. The internet program provided to patients included photos and videos of the exercises, as well as information on relevant musculoskeletal anatomy, whiplash injury mechanisms, neurophysiological and neurobiological pain education, and strategies for managing neck pain relapse. The web-based system also included an explanation and justification for why participants should perform their neck exercises. Patients could contact their physiotherapist if necessary. Exercises were adjusted individually according to each individual’s physical conditions [33].

### Cost-Effectiveness Analysis

#### Measurements

Participants completed questionnaires via a research website at baseline and at the 3- and 15-month follow-ups. A small number of participants opted to complete the questionnaires on paper instead. We used a complete case analysis consisting of 56 participants in the NSEIT group and 58 participants in the NSE group.

The primary health outcome indicator for the cost-effectiveness analysis was change in HRQoL, calculated based on measurements using EQ-5D-3L [37] (scores ranging from –0.594 to 1, where 1=full health) and the EQ-VAS thermometer (0-100 mm, where 100=full health) [32,35]. The primary outcome of this study was the incremental cost-effectiveness ratio (ICER; a cost-to-effect ratio of NSEIT in relation to the control treatment, NSE) from a Swedish societal perspective, encompassing the costs of interventions, additional health care, drugs, and production losses. The effect was calculated based on the participants’ rating of (HRQoL), using the EuroQoL group’s EQ-5D-3L instrument [38].

#### Cost Measure

The intervention cost included charges for the physiotherapist per session. Additional health care service costs included costs associated with physician visits, visits to other physicians, visits to physiotherapists other than those providing the interventions, visits to psychologists or counselors, visits to chiropractors or similar practitioners, visits to a pain clinic for diagnosis or treatment, or visits to emergency care. These costs were considered additional health care costs and were also determined from the patient questionnaire at 3 and 15 months. Out-of-pocket costs were not available (cost details are provided in Table S2 in Multimedia Appendix 1). These, along with medicine costs, were considered direct costs.

The medicine cost included medications for the management of WAD symptoms. Patients reported the number of tablets taken daily, weekly, monthly, and annually. An average was calculated to estimate the total medication intake over the 15 months. The unit cost was based on the county council price list for the trial region in 2019.

The intervention costs were derived from the number of visits to physiotherapists reported in the patient questionnaire. The 2019 cost per physiotherapy visit for the trial regions was used in the analysis. Details of other unit costs are presented in the table, along with the respective resources.

Indirect costs mainly included sick leave due to WAD, activity support due to WAD, and unemployment support if the patient had lost their job due to WAD injury or accident. These data were self-reported by the patients. Patients reported receiving sick leave benefits, which were generally about 80% of the lost income for a person registered under Swedish social insurance. The total income was based on the median salary in Sweden, according to Statistics Sweden. Similarly, the costs of activity support and unemployment support were derived from percentages reported by patients. The national costs were obtained from the Social Security report and the Swedish Unemployment Agency. Discounting was unnecessary because the follow-up period was limited to 1 year after the intervention. Most health care costs were incurred in 2019, so the European Central Bank’s average conversion rate [39] was used to convert the ICER from Swedish krona (SEK) per point to euros per point.

### Statistical Methods

A statistical comparison between the 2 groups was conducted using the chi-square test and 2-tailed *t* tests. Additionally, univariate analysis of variance was conducted with adjustment for baseline values for the groups to estimate the adjusted mean HRQoL scores for both groups. The 15-month HRQoL was included in the analysis because the intervention effects were expected to be greater at 15 months, whereas the 3-month HRQoL could be less affected in patients with chronic WAD patients. The study included only complete cases who followed through to the 15-month follow-up. The statistical analysis, following intention-to-treat principles, was conducted using SPSS (version 29; IBM Corp). The ICER was calculated as the ratio of the difference in costs to the mean difference in HRQoL using unadjusted analysis. The ICER was interpreted based on its value. To assess the robustness of the findings, a probabilistic sensitivity analysis was conducted using a Monte Carlo simulation model [40] with 1000 iterations. The costs followed gamma distributions, and utilities followed beta distributions (details in Table S3 in Multimedia Appendix 1). If the intervention was more costly and more effective, its cost-effectiveness depended on the decision makers’ willingness to pay (WTP), to be SEK 500,000 point (SEK 1=US $0.10 as of October 6, 2026) [41]. At the same time, if it was less costly but less effective, the cost-effectiveness depended on the willingness to accept costs for lower effectiveness [42]. A 1-way sensitivity analysis was conducted to assess the effect of various costs incurred by the groups on the base case ICER, to test their isolated effects. Intervention cost, medicine cost, social welfare cost, and health care cost incurred by both groups were included as parameters. Changes of 20% in the base costs were tested in the sensitivity analysis. The tornado diagram was developed based on the output.

### Ethical Considerations

The study received approval from the regional ethical review board in Linköping, Sweden (Dnr 2016/135–31, 2016/526–32, and 2017/45–32), and adhered to the principles of the Declaration of Helsinki. The trial was registered with ClinicalTrials.gov (NCT0302281212) prior to data collection. Written informed consent was obtained from all participants. The data were anonymized, and the analysis was conducted at the group level without any connection to an individual participant. No compensation or any monetary and/or in-kind support was provided by the researchers to the study participants.

## Results

### Overview

A total of 140 participants were randomly assigned to either the NSEIT (n=70) or NSE (n=70) groups. Out of these, total 137 were included in the study NSEIT (n=69) or NSE (n=68) groups. There were 78.8% (108/137) women, with 54 women each in the NSEIT and NSE groups. The mean age was 40.36 (SD 11.3) years, with an age range of 19-64 years. The groups did not differ in any baseline variables, including the EQ-5D scores. The baseline analysis and participant characteristics are presented in Table 1. There was no correlation between age or biological sex and health outcomes (*P*>.05]. No individuals in the NSEIT group received unemployment support, compared with 1 in the NSE group (Table S1 in Multimedia Appendix 1). Two individuals from the NSEIT group received parental support, whereas none from the NSE group did at the beginning of the study. An equal number—3 individuals—were receiving activity support (earlier disability support) due to WAD in both groups at the beginning (Table S1 in Multimedia Appendix 1). At the 15-month follow-up, 56 (81.1%) participants in the NSEIT group and 58 (85.2%) participants in the NSE group were successfully followed up.

**Table 1:** Background and baseline variables of the participants in neck-specific exercise with internet support (NSEIT) and neck-specific exercise (NSE) interventions and cost data at 15 months.

			NSEIT (n=69)		NSE (n=68)
**Sex, n (%)**					
	Female	54 (78.3)		54 (79.4)	
	Male	15 (21.7)		14 (20.6)	
**Education level, n (%)**					
	High school	30 (43.5)		38 (55.9)	
	University	34 (49.3)		26 (38.2)	
	Other	5 (7.2)		4 (5.9)	
**Main source of income, n (%)**					
	Permanent work	51 (73.9)		54 (79.4)	
	Temporary work	2 (2.8)		2 (2.9)	
	Hourly work	7 (10.1)		4 (5.9)	
	Salary allowance	1 (1.4)		0	
**Income reduction due to WAD^a^** **, n (%)**					
	Yes	38 (55.9)		34 (50.0)	
	No	30 (44.1)		34 (50.0)	
**Take medicine to alleviate your neck pain, n (%)**					
	Yes	57 (82.6)		56 (83.6)	
Number of visits to any health care provider in the last 6 months for neck pain, mean (SD)			20.4 (16.35)		15.75 (17.71)
**Health outcomes, mean (SD)**					
	EQ-5D	0.563 (0.289)		0.637 (0.217)	
	EQ-5D-VAS	58.13 (18.5)		59.46 (16.34)	
**Costs at 15 months (SEK^b^), mean (SD)**					
	Intervention cost	5956.21 (1088.41)		27,511.63 (9127.9)	
	Health care services cost	6129.51 (16,485.39)		11,252.80 (43,634.16)	
	Medicines cost	796.64 (2949.50)		640.88 (1344.44)	
	Social welfare cost	—^c^		682.33 (3921.41)	
	Total cost	12,882.36 (17,578.54)		40,087.64 (46,147.36)	

^a^WAD: whiplash-associated disorders.

^b^SEK 1=US $0.10 as of October 6, 2026.

^c^No social welfare support was reported by participants.

### EQ-5D and EQ-VAS

After 15 months, both groups reported improvements in EQ-5D scores and EQ-VAS scores (Figures 1 and 2). However, *t* test results showed that there was no significant difference in EQ-5D scores between NSEIT and NSE (t_106_=–1.064; *P*=.29; 95% CI –0.142 to 0.43). The EQ-5D score was also calculated by adjusting for the baseline score. The difference between NSEIT and NSE was –0.025 (95% CI –0.106 to 0.056) and was statistically nonsignificant. (*P*=.55; Table 2). Similarly, for the EQ-VAS score, no significant difference was recorded (t_106_=–1.606; *P*=.11; 95% CI –13.5 to 1.42).

**Figure 1:**
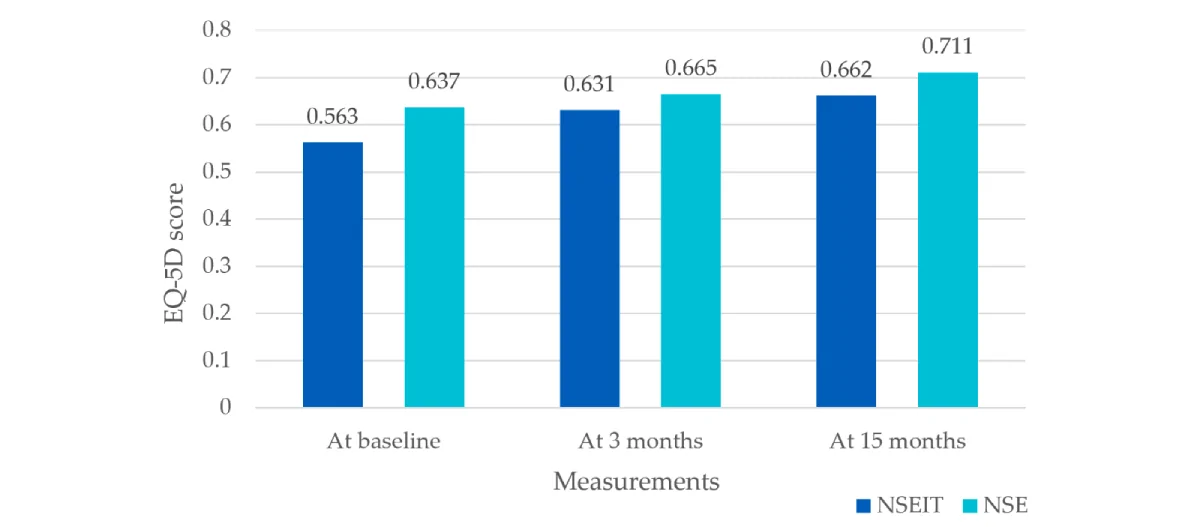
EQ-5D scores. NSE: neck-specific exercise; NSEIT: neck-specific exercise with internet support.

**Figure 2:**
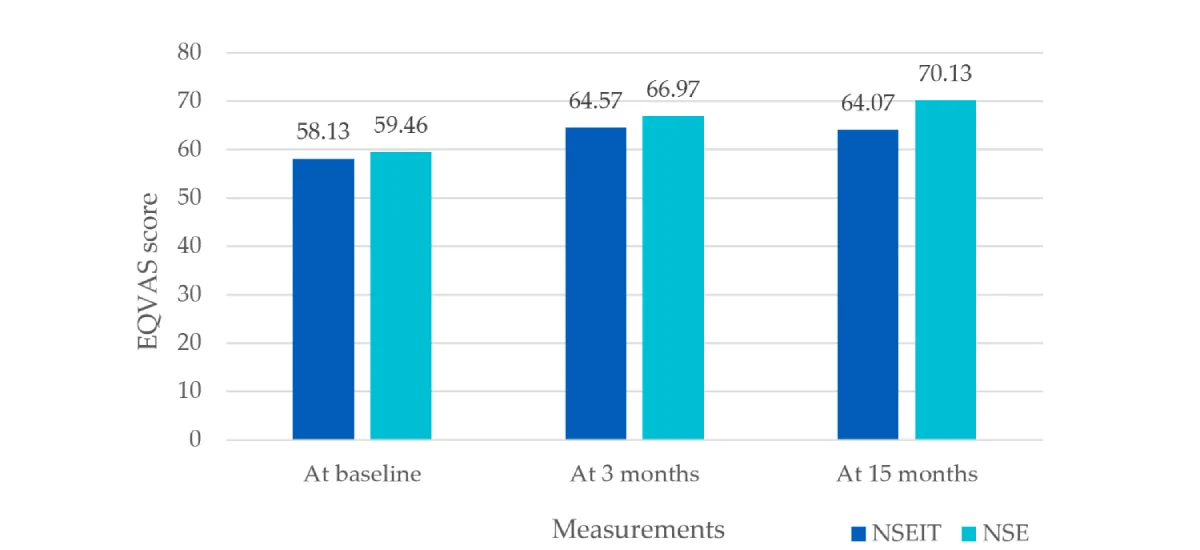
EQ-5D-VAS scores. NSE: neck-specific exercise; NSEIT: neck-specific exercise with internet support.

**Table 2:** Base case incremental cost-effectiveness ratio (ICER) with unadjusted and adjusted effects (EQ-5D).

			NSEIT^a^ (n=56)		NSE^b^ (n=58)	
**Unadjusted analysis**						
	Total cost (SEK^c^)	12,882.36		40,087.64		
	HRQoL^d^, mean (SD)	0.662 (0.256)		0.711 (0.229)		
	Incremental cost (SEK; NSEIT–NSE)	–27,205.28		—^e^		
	Incremental effect (HRQoL; NSEIT–NSE)	–0.049		—		
	ICER (SEK/point)	555,209.80				—
	ICER (Euro/point)^f^	52,927.53				—
**Adjusted analysis**						
	Total cost (SEK)	12,882.36		40,087.64		
	HRQoL, mean (SD)	0.675 (0.029)		0.699 (0.029)		
	Incremental cost (SEK; NSEIT – NSE)	–27,205.28		—		
	Incremental effect (HRQoL; NSEIT – NSE)	–0.025		—		
	ICER (SEK/point)	108,8211.2				—
	ICER (Euro/point)	103,737.96				—

^a^NSEIT: neck-specific exercise with internet support.

^b^NSE: neck-specific exercise.

^c^SEK 1=US $0.10 as of October 6, 2026.

^d^HRQoL: health-related quality of life.

^e^Not applicable.

^f^Based on the average euro conversion rate in 2019 (€1=SEK 10.49).

### Costs

The total cost was calculated as the sum of intervention cost, additional health care services cost, drug cost, and the cost of production loss. Figure 3 illustrates the comparative costs of both interventions. At the 15-month follow-up, the total mean costs calculated for NSEIT and NSE were SEK 12,882 (SD 17,578.54) and SEK 40,087 (SD 46,147.36), respectively (Table 2).

**Figure 3:**
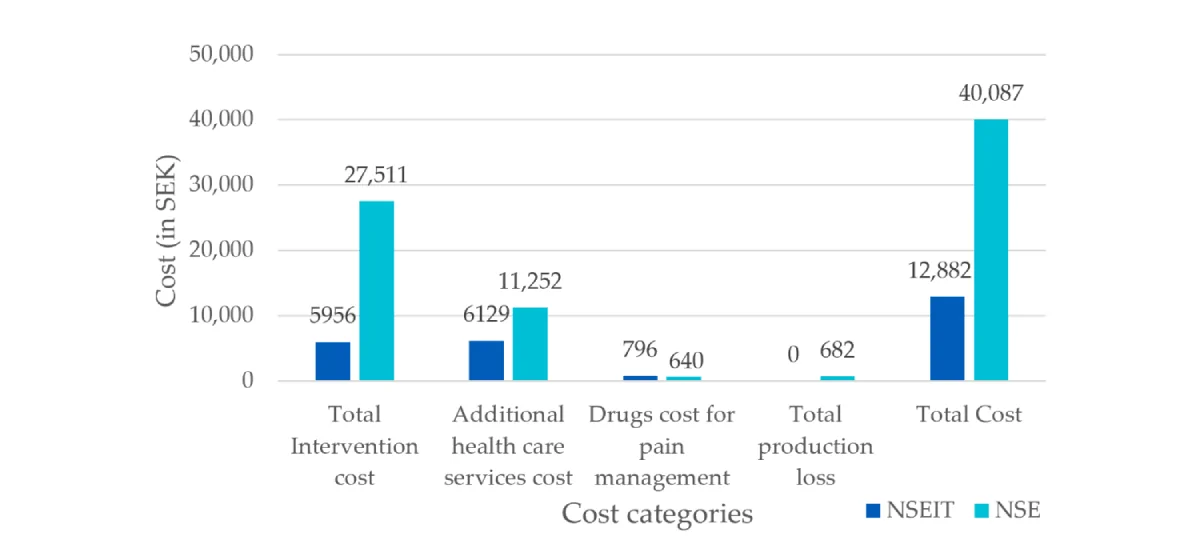
Comparison of costs incurred (in SEK) by 2 groups. NSE: neck-specific exercise; NSEIT: neck-specific exercise with internet support.

### Cost-Effectiveness Analysis

The ICER for the EQ-5D score was SEK 555,209.80 per point (Table 2) using unadjusted effects. The estimated mean cost per person at the 15-month follow-up was SEK 12,882.36 for the NSEIT group and SEK 40,087.64 for the NSE group. The NSEIT intervention was not cost-effective compared with the current NSE practice, despite yielding lower total costs.

### Sensitivity Analysis

The cost-effectiveness plane, as determined through sensitivity analysis, is positioned primarily in the southwest quadrant, indicating a positive ICER value (Figure 4). Cost effectiveness acceptability curve (CEAC) shows that at the threshold of WTP (SEK 500,000 per point), there is a moderate (0.57) probability of the intervention being cost-effective with loss of HRQoL. One-way sensitivity analysis [40] is illustrated in the tornado diagram (Figure 5). The health care costs of individuals under NSE and NSEIT are the ones that chiefly impact the ICER. In contrast, the intervention cost of NSEIT is the least impactful factor in impacting the ICER.

**Figure 4:**
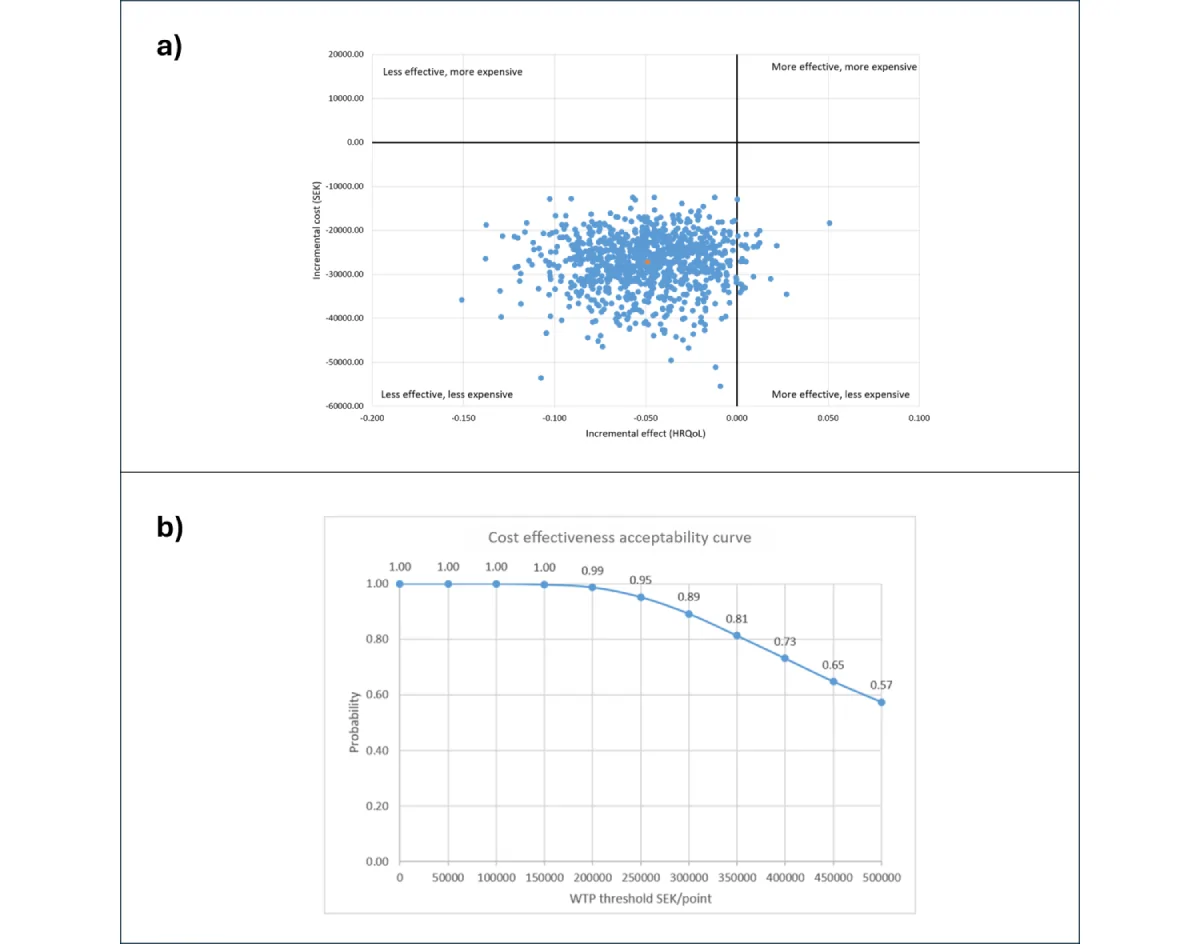
(a) Cost-effectiveness planes from a societal perspective for differences in health-related quality of life (HRQoL; EQ-5D). (b) Cost-effectiveness acceptability curves from a societal perspective: willingness to pay (WTP) in SEK.

**Figure 5:**
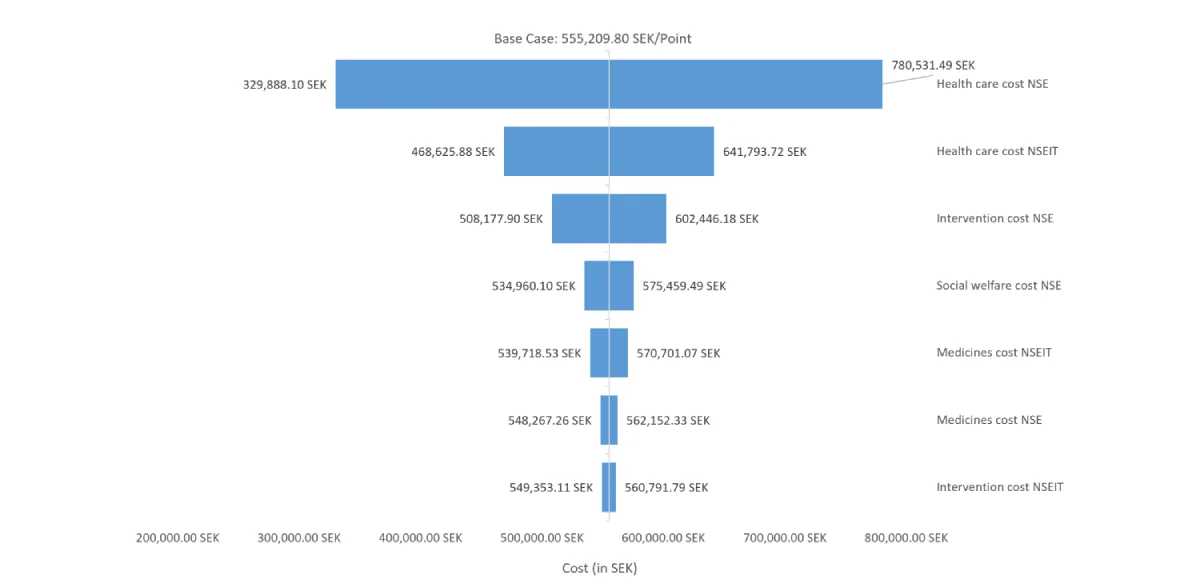
Tornado diagram of 1-way sensitivity analysis of neck-specific exercise with internet support (NSEIT) in comparison with neck-specific exercise (NSE).

## Discussion

### Principal Findings

Both interventions showed gains in EQ-5D scores and improvements in EQ-5D VAS scores (NSEIT: from 58.13 to 64.07; NSE: from 59.46 to 70.13). The difference between the groups in HRQoL was not statistically significant. Earlier studies of the same intervention at 3 and 15 months found noninferiority of NSEIT to NSE on EQ-5D and other health outcomes, indicating the closeness of health outcomes and confirming the findings of this study [30,33]. An earlier study assessing NSE and NSE with a behavioral approach found NSE to be cost-effective from a societal perspective [31], a finding that was consistent with this study.

The study showed that total intervention costs for NSEIT were significantly lower than those for NSE in patients with chronic WAD because patients receiving NSEIT had fewer in-person visits with a physiotherapist than those receiving NSE. While mean medicine costs were higher in the NSEIT group, other health care costs were also lower in the NSEIT group. The health care services included visits to general practitioners, physicians, counselors, chiropractors, pain clinics, and emergency departments. NSEIT was less costly. It also had lower HRQoL compared with NSE, resulting in decremental cost-effectiveness, which was consistent with an earlier study assessing a similar blended physiotherapy intervention [24].

The present study is the first to investigate the cost-effectiveness of an internet-based intervention for chronic WAD. The calculated ICER for NSEIT and NSE is SEK 555,209.80 per point, which lay in the southwest quadrant of the cost-effectiveness plane, where both the incremental cost and the incremental effect were negative [43], leading to a decremental ICER.

One explanation for NSEIT not being cost-effective compared with NSE might be that the difference in effectiveness between the interventions was minimal. Researchers for the same project previously recorded that the self-reported compliance of the NSE group was significantly higher than that of NSEIT [33]. Earlier studies have demonstrated that adherence to eHealth interventions and their intended use can be challenging [44-46], which may have been the case with NSEIT as well. The studies recommended using a persuasive design for internet-based interventions to ensure adherence and intended use. The interventions could be improved holistically to enhance adherence and use, ultimately improving overall effectiveness. The HRQoL and other health outcomes of NSEIT and NSE did not differ statistically [33].

The cost-effectiveness plane spans the southwest quadrant, indicating a high cost difference with negative incremental health outcomes. Cost Effectiveness Acceptability Curve (CEAC) indicated that at a WTP of SEK 500,000 per point, there was a moderate (0.57) probability that the intervention was cost-effective. This implied that society might save by choosing NSEIT, as the monetary value of lost health outcomes was less than the cost savings. This also implied that the difference in health outcomes between NSEIT and NSE was minimal and that, from a societal perspective, NSEIT may be considered for implementation in resource-constrained settings. Sensitivity analysis found that additional health care costs for individuals receiving NSE and NSEIT had the greatest impact on the ICER, followed by the intervention cost of NSE. This also suggested that individuals’ overall health and well-being may be considered before they receive IT-based or in-person interventions. This could be due to the high cost of health care services in general.

Another explanation for the smaller difference in effectiveness is that the NSEIT group had lower EQ-5D-3L scores at baseline and more sick leave, although the difference was not statistically significant (*P*=.29). In addition to the potential for reduced compliance, this could have contributed to the continued high sickness level in the group, thereby affecting the EQ-5D scores at the end of 15 months. Individuals in the NSEIT group also reported greater medication use, especially at baseline. The cost of medications was higher for NSEIT than for NSE. This might be because of other symptoms related to chronic WAD, like pain, anxiety, stress, dizziness, tiredness, and, in general, poor well-being. Such symptoms could also have contributed to the EQ-5D scores at the 15-month follow-up.

### Limitations

The cost-effectiveness analysis undertaken in this study should be interpreted with caution, given certain limitations of the dataset and the study’s context. First, the study relied on self-reported data from patients regarding health care use. Such self-reported data often have recall bias, as individuals may over- or underreport actual health care use. The costs were estimated based on the number of visits to health care services. The number of these visits could have been over- or underestimated.

Additionally, with a small sample size, costs and EQ-5D scores may vary widely. The study was conducted in limited geographic areas. A larger study with a higher sample size across wider geographic areas could have a better opportunity for cost-effectiveness analysis. This, along with patient attrition, may have yielded an ICER that is sensitive to small analytic changes. This limits the reliability of the findings. Going forward, secondary register data for each patient could be helpful in cost estimation.

In cost-effectiveness analysis, presenteeism is one of the key costs in productivity estimates, especially when investigating chronic pain and function-related disorders. Presenteeism costs were not included due to the unavailability of data. This may also have affected ICER calculations and, to some extent, increased the costs of both treatment modalities.

Apart from these limitations, it must be understood that while the study may be generalizable within the Swedish health care system, with certain regional variations in costs. The results may have limited generalizability to global health care systems that operate under different cost structures, compared with the publicly funded Swedish health care system.

### Conclusions

We found that the NSEIT intervention—an eHealth intervention for chronic WAD—was less costly and less effective when compared with NSE. Health care costs associated with both interventions affected the ICER; therefore, patients’ overall well-being should also be considered when treating WAD. Even though the CEAC showed that the cost savings from NSEIT outweighed the loss of health outcomes, the reliability of the findings is limited by the exclusion of presenteeism costs, patients’ self-reported health care use, and the small difference in health outcomes. Further research may include register-based data on health care use and sick leave to improve precision in cost calculations. Collecting adherence data through the web-based system is also recommended to avoid recall bias in evaluation.

## Appendix

Multimedia Appendix 1 Additional cost details.

Multimedia Appendix 2 CHEERS 2022 checklist.

## Abbreviations

CEAC: Cost effectiveness acceptability curve
CHEERS: Consolidated Health Economic Evaluation Reporting Standards
HRQoL: health-related quality of life
NSE: neck-specific exercise
NSEIT: neck-specific exercise with internet support
WAD: whiplash-associated disorders
WTP: willingness to pay

## Data Availability

Anonymized data may be shared upon reasonable request from the corresponding author subject to ethics approval.
