# Supplementary material for: Neck-Specific Exercises With Internet Support Versus Standard Neck-Specific Exercise for the Treatment of Chronic Whiplash-Associated Disorders: Cost-Effectiveness Analysis
Source: J Med Internet Res. 2026 Oct 28;28:e88832. doi: 10.2196/88832 (PMC13755776; doi:10.2196/88832)
Supplement: Supplementary file 1 [file jmir_v28i1e88832_app1.docx]

**Table S1.** Baseline variables.

|  | | NSEIT | NSE |
| --- | --- | --- | --- |
| **Number of visits to various health care professionals for WAD, mean (SD)** | | | |
|  | Physician Private and/or Health centre visits | 3.42 (4.038) | 1.63 (1.691) |
|  | Physiotherapist Private and/or Health centre visits | 6.42 (7.919) | 8.26 (11.621) |
|  | Physician at Emergency services visits | 1.1 (1.338) | 2.19 (5.269) |
|  | Other doctors at hospital visits | 0.47 (1.246) | 0.26 (0.689) |
|  | Physiotherapist at Hospital visits | 0.25 (0.577) | 0.33 (0.637) |
|  | Doctor at work's health support visits | 0.88 (2.527) | 0.05 (0.218) |
|  | Physiotherapist at work's health support visits | 1.88 (3.257) | 0.83 (2.289) |
|  | Counsellor visits | 2.65 (3.725) | 2.65 (6.692) |
|  | Psychologist visits | 2.32 (2.587) | 4.2 (8.467) |
|  | Chiropractor or similar visits | 0.35 (0.862) | 0 |
|  | Diagnosis at pain clinic visits | 0 | 0.95 (4.259) |
|  | Individual treatment at pain clinic visits | 3.41 (4.331) | 2.67 (6.658) |
|  | Group treatment at pain clinic visits | 0 | 0 |
|  | Other | 0 | 0 |
|  | Use of medicines for neck pain relief, yes, n, (%) | 57 (82.6) | 56 (83.6) |
| **Number of tablets in the past year, mean (SD)** | | | |
|  | Paracetamol | 360 | 0 |
|  | Other anti-inflammatory tablets | 30 (28.28) | 17.5 (17.68) |
|  | Anti-inflammatory gel (applied) | 0 | 2 (1.41) |
|  | Mild opioids | 6.25 (5.96) | 4 () |
|  | Strong opioids | 3 (2.83) | 0 |
|  | Antidepressants | 3 | 0 |
|  | Antiepileptic | 0 (0) | 0 |
|  | Muscle relaxants | 15.33 (12.86) | 17 (9.90) |
|  | Natural medicines | 0 | 0 |
|  | Other medicines | 100 | 0 |
|  | One or more sick leaves of less than 14 days in the past year because of WAD | 32. (46.4) | 26 (38.2) |
| **Social care benefits, n (%)** | | | |
|  | Sick pay for WAD | 4 (6) | 7(10) |
|  | Sick pay for other health issues | 1 (1) | 1 (1) |
|  | Activity support for WAD | 3 (4) | 3 (4) |
|  | Activity support for other health issues | 2 (3) | 1 (1) |
|  | Parental allowance | 2 (3) | 0 |
|  | Educational allowance | 6 (9) | 5 (7) |
|  | Unemployment support | 0 | 1 (1) |

**Table S2.** Unit costs.

| Resource | | Unit cost | Source |
| --- | --- | --- | --- |
| **Intervention cost** | | | |
|  | One physiotherapist session | 1502 | Price list for region 2019 |
| **Additional health care services** | | | |
|  | Physician | 2275 | Price list for region 2019 |
|  | Other doctors | 8773 | Price list for region 2019 |
|  | Physiotherapist | 1932 | Price list for region 2019 |
|  | Psychologist and/or Counsellor | 2485 | Price list for region 2019 |
|  | Chiropractor or similar | 1213 | Price list for region 2019 |
|  | Visit to a pain clinic for diagnosis or treatment | 2250 | Price list for region 2019 |
|  | Visits to emergency care | 11378 | Price list for region 2019 |
| **Drugs for pain management** | | | |
|  | Paracetamol | 2 | Swedish Medicines Agency 2021 |
|  | Other anti-inflammatory tablets | 1.75 | Swedish Medicines Agency 2021 |
|  | Anti-inflammatory gel | 1.83 | Swedish Medicines Agency 2021 |
|  | Mild opioids | 3.24 | Swedish Medicines Agency 2021 |
|  | Strong opioids | 3.84 | Swedish Medicines Agency 2021 |
|  | Antidepressants | 0.99 | Swedish Medicines Agency 2021 |
|  | Antiepileptic | 4.38 | Swedish Medicines Agency 2021 |
|  | Muscle relaxants | 1.58 | Swedish Medicines Agency 2021 |
| **Production loss** | | | |
|  | Sick pay | 28480 | 80% of the salary. median salary taken from SCB |
|  | Activity support | 11944 | As per the social security report 2024 |
|  | Unemployment support | 26400 | As per Public Employment Service |

**Table S3.** Parameters and distribution for PSA.

| Variables | | Distribution | Mean | SE |
| --- | --- | --- | --- | --- |
| **Cost** | | | | |
|  | Intervention cost NSE | Gamma | 27511.63 | 1178.407 |
|  | Health care cost NSE | Gamma | 11252.8 | 5633.146 |
|  | Medicines cost NSE | Gamma | 640.88 | 173.567 |
|  | Social welfare cost NSE | Gamma | 682.33 | 506.252 |
|  | Intervention cost NSEIT | Gamma | 5956.21 | 142.916 |
|  | Health care cost NSEIT | Gamma | 6129.51 | 2164.638 |
|  | Medicines cost NSEIT | Gamma | 796.64 | 387.289 |
| **Utilities** | | | | |
|  | QALY NSEIT | Beta | 0.662 | 0.035 |
|  | QALY NSE | Beta | 0.711 | 0.031 |
